# Rapid Eye Movements in Sleep Furnish a Unique Probe into the Ontogenetic and Phylogenetic Development of the Visual Brain: Implications for Autism Research

**DOI:** 10.3390/brainsci15060574

**Published:** 2025-05-26

**Authors:** Charles Chong-Hwa Hong

**Affiliations:** 1 Patuxent Institution, Correctional Mental Health Center—Jessup, Jessup, MD 20794, USA; charleschhong@gmail.com; 2 Department of Psychiatry and Behavioral Sciences, The Johns Hopkins Hospital, Baltimore, MD 21205, USA

**Keywords:** rapid eye movements in sleep, saccadic eye movements, multisensory integration, visual perception, neurodevelopment, functional MRI, autism spectrum disorders, animal model

## Abstract

With positron emission tomography followed by functional magnetic resonance imaging (fMRI), we demonstrated that rapid eye movements (REMs) in sleep are saccades that scan dream imagery. The brain “sees” essentially the same way while awake and while dreaming in REM sleep. As expected, an event-related fMRI study (events = REMs) showed activation time-locked to REMs in sleep (“REM-locked” activation) in the oculomotor circuit that controls saccadic eye movements and visual attention. More crucially, the fMRI study provided a series of unexpected findings, including REM-locked multisensory integration. REMs in sleep index the processing of endogenous visual information and the hierarchical generation of dream imagery through multisensory integration. The neural processes concurrent with REMs overlap extensively with those reported to be atypical in autism spectrum disorder (ASD). Studies on ASD have shown atypical visual processing and multisensory integration, emerging early in infancy and subsequently developing into autistic symptoms. MRI studies of infants at high risk for ASD are typically conducted during natural sleep. Simply timing REMs may improve the accuracy of early detection and identify markers for stratification in heterogeneous ASD patients. REMs serve as a task-free probe useful for studying both infants and animals, who cannot comply with conventional visual activation tasks. Note that REM-probe studies would be easier to implement in early infancy because REM sleep, which is markedly preponderant in the last trimester of pregnancy, is still pronounced in early infancy. The brain may practice seeing the world during REM sleep in utero before birth. The REM-probe controls the level of attention across both the lifespan and typical-atypical neurodevelopment. Longitudinal REM-probe studies may elucidate how the brain develops the ability to “see” and how this goes awry in autism. REMs in sleep may allow a straightforward comparison of animal and human data. REM-probe studies of animal models of autism have great potential. This narrative review puts forth every reason to believe that employing REMs as a probe into the development of the visual brain will have far-reaching implications.

## 1. Introduction

Autism spectrum disorder (ASD) is a highly genetically heterogeneous [1] lifelong neurodevelopmental disorder. Whereas ASD affects 1 out of 36 children in the United States [2], 1 of 5 infant siblings of children with ASD later received an ASD diagnosis [3]. Early detection and intervention can improve the deficits of ASD and long-term outcomes. The Infant Brain Imaging Study (IBIS) Network conducted magnetic resonance imaging (MRI) studies of infants at high risk for ASD, typically during natural sleep, and showed that brain imaging markers from the first year of life predicted later ASD diagnosis among infant siblings [4,5]. Moreover, their recent infant sibling study recognized aberrant visual brain development as a potential MRI marker of ASD [6], which prompted this review paper. To improve the accuracy of early detection of ASD, stratify ASD patients, and identify a subtype with aberrant visual brain development [4], I suggest the addition of timing rapid eye movements (REMs) in sleep. REMs under closed eyelids can be timed from video recordings in functional MRI (fMRI) studies (see Supplementary Materials).

The revised diagnostic criteria (Diagnostic and Statistical Manual of Mental Disorders, Fifth Edition) for autism include atypical sensory experience as a core diagnostic feature, along with social deficits, restricted interests, repetitive behaviors, and resistance to changes [7]. Atypical sensory experiences affect most (>70%) autistic individuals in all sensory modalities and potentially serve as early diagnostic markers; the neural underpinnings of atypical sensory experiences and processing have been theorized [8,9]. Studies on autism showed alterations across the cerebral cortex, especially in the primary visual cortex (V1), emerging early in infancy and persisting across development [5,6,10] (for reviews, [8,11]). The “sensory-first account of autism” proposed developmental cascades of differences in visual sensory processing into autistic symptoms [11,12]. Atypical multisensory integration in autism has also been demonstrated, and a cascading effect thereof on the development of atypical social communication has been suggested [13,14,15,16,17,18,19].

Crucially, our event-related fMRI studies (with the events being REMs identified from video recordings) demonstrated that visual processing and multisensory integration are time-locked to REMs in sleep (“REM-locked”), i.e., concurrent with REMs [20,21]. Note that we studied brain activity time-locked to REMs in sleep, not baseline brain activity during REM sleep. Note that REMs in sleep are saccades, as explained below. Employing REMs in sleep as a probe (“REM-probe”) may help elucidate the neural underpinnings of the atypical visual sensory processing and multisensory integration seen early in infancy in autistic individuals. REM-probe studies would be easier to implement in early infancy because REM sleep, which is markedly preponderant in the last trimester of pregnancy, gradually reduces, but is still pronounced in early infancy [22,23].

To sample visual sensory data and enable waking visual perception, the eyes move rapidly from one fixation point to the next and fixate briefly to sample visual information [24]. These scanning, ballistic REMs are called saccades and occur at a rate of 3–4 per second in both wakefulness and sleep [25]. The brain automatically chooses points to scan that provide salient or precise information, such as on another’s eyes and mouth. We demonstrated with positron emission tomography (PET) [26], and then with fMRI [20], that REMs in sleep are saccades that scan dream imagery. In what is widely regarded as the standard textbook on eye movements, REMs in sleep are considered saccades [27]. What about animals? Recently, an animal study demonstrated coupling of the head direction system with REMs, both in sleeping and awake mice, and confirmed that REMs in sleep are saccades that scan the virtual world of dreams, akin to saccadic eye movements in wakefulness that scan the environment during exploration [28]. It is relevant to autism research that REMs in sleep are saccadic scans. Atypicality in saccadic eye movements, visual attention, and visual–motor integration, as reported in children with ASD, may be a prodromal sign of a neurodevelopmental disorder having cascading effects on social development; low-cost, non-invasive eye tracking may become a tool for early detection of and intervention in ASD [29]. For example, a US Food and Drug Administration-authorized eye-tracking device measuring social visual engagement predicted expert diagnoses of autism with high specificity and sensitivity in toddlers (aged 16–30 months) [30]. REM-probe studies of infant siblings of children with autism may distinguish typical and atypical development of dreaming and waking consciousness, particularly visual perception.

Saccades actively sample the world to infer “the signal source (world)” [31]. The brain, enclosed inside the skull, samples visual and non-visual sensory signals from the outside world and makes probabilistic knowledge-driven inferences to ascertain their source [31,32,33,34]. This view builds on the insight of von Helmholtz, who, in the 19th century, posited that perception is an internal, inferential, and constructive process [35]. In this conception, the brain generates a model of the world “hidden” behind sensory data, and this inference machine runs in essentially the same way when using endogenous sensory data, i.e., when dreaming [20,26,36,37,38]. The process of generating visual percepts is constrained only by sensory signals from the environment when awake [34]. This view is in line with the “controlled hallucination” view of waking perception [39].

Likewise, we do not perceive the body merely because it is there, but rather because the brain is able to generate a model of the bodily self from interoceptive signals arising from the body [40]. Multimodal sensory integration is essential for generating a bodily self-model during wakefulness [41,42,43] and apparently also in dreaming [25].

Both in wakefulness [41] and in dreaming [44], the virtual body is situated at the center of virtual reality, not only as an observer but also as an agent interacting with the world, including other persons [25]. Our fMRI findings [20] fit well with the view of the function of consciousness, namely, generation of the world model, with the self-model at its center for adaptive interaction of the self with the world in simulations [25,41,45,46]; this has been described as “[a highly] advanced, user-friendly interface design” and “a wonderfully efficient control device” [45]. This view of self–world interaction is relevant to the atypical social communication and interactions of autistic individuals.

Hobson proposed that this body–world interaction is simulated in REM sleep in the uterus to promote the survival of our physical body in the physical world after birth [23,47]. REM sleep is markedly preponderant in the last trimester of pregnancy [22,23]. In Hobson’s “protoconsciousness theory”, REM sleep plays a foundational role in the development of consciousness starting in the third trimester [23]. This is in line with a recent review showing that consciousness may arise in the third trimester [48]. We may practice walking during REM sleep in utero before we actually learn to walk after birth [47]. Likewise, the brain may practice seeing the world during REM sleep in utero before birth. This is relevant to autism research because “ASD begins in prenatal life”, and “most ASD risk genes are expressed prenatally in many ASD-relevant brain regions” [49].

As expected, our fMRI study of (video-timed) REMs in sleep [20] revealed REM-locked activation in the oculomotor circuit that controls saccadic eye movements and visual attention. More crucially, our fMRI study provided a series of unexpected, surprising findings, revealing some of the fundamental processes of visual processing that underlie our experience of the world in both dreaming and wakefulness [34]. The surprising findings of our fMRI study (expounded on below) [20] prompted Allan Hobson to invite me and Karl Friston to publish a paper [34]. Our fMRI findings [20] provide empirical support for predictive coding theory, as well as for the “protoconsciousness theory” [34]. We found that REMs in sleep index the processing of endogenous visual information and hierarchical generation of the world model (particularly visual percepts) through multisensory–motor integration [34]. REMs in sleep (i.e., saccades) not only scan but also generate dream visual imagery, as mandated by “active inference” under predictive coding [34,50].

This is a non-systematic, narrative review. I used Google Scholar to search relevant papers. I selected papers cited in high numbers, papers published in prestigious journals, and meta-analyses. References in those selected papers helped me make other selections.

This review aims to excite and inform researchers regarding the great potential of employing REMs in sleep as a probe of the phylogenetic and ontogenetic development of consciousness (particularly visual percepts), including its aberrant development in autism, starting from infancy. Our fMRI studies revealed REM-locked peak activation sites [V1 and the thalamic reticular nucleus (TRN), claustrum, cholinergic system, retrosplenial cortex in the right hemisphere (RSC-Rt), and bed nucleus of the stria terminalis (BNST)] and REM-locked neural processing [control of saccadic eye movements/visual attention, processing of visual information, multisensory–motor integration, and anticorrelation between the default mode network (DMN) and visual processing network] [20,21]. Do these REM-locked peak activation sites and processes overlap with those reported to be atypical in autistic individuals and animal models of autism? This review shows that the overlap is extensive, as described below.

## 2. fMRI Study of the Neural Correlates of REMs in Sleep

We studied precise temporal changes in brain activities time-locked to REMs. Instead of comparing non-REM sleep with REM sleep, or tonic (REM sleep with no REMs) with phasic REM sleep (REM sleep with REMs), using a block design, we used event-related fMRI [20,21]. Notably, REM-locked brain activity is distinct from baseline activity during phasic REM sleep [21,25]. Taking into account this important subtlety will improve understanding of the utility of REMs in sleep as a probe, given the high capacity to localize REM-locked peak activities and statistical efficiency.

### 2.1. Summary of the Findings

**Figure 1:**
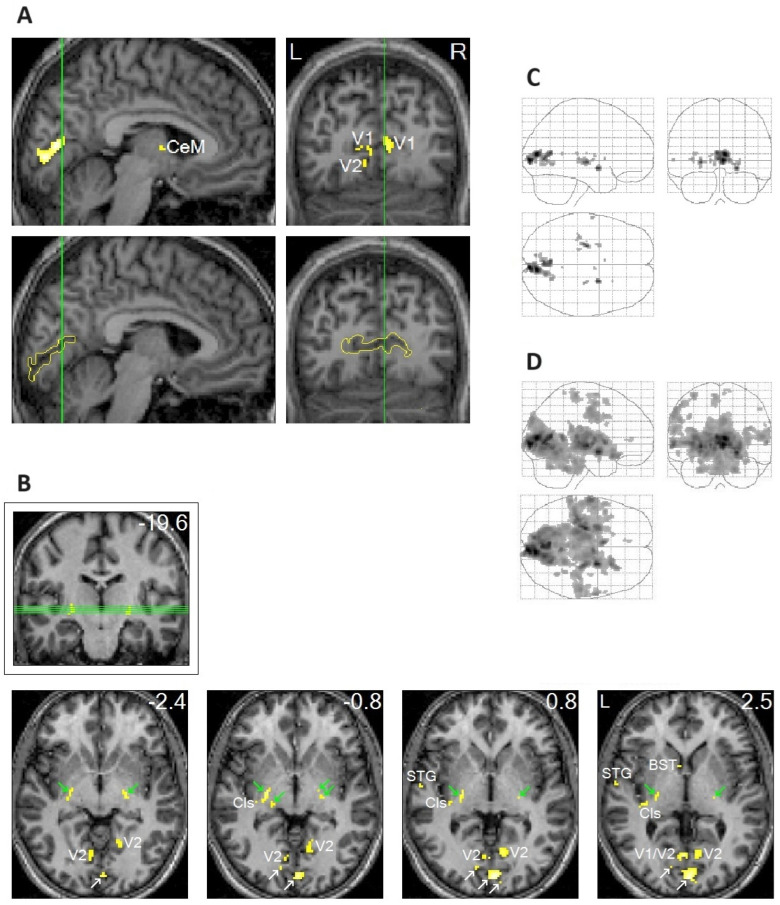
Peak rapid eye movement (REM)-locked activation in adults (functional magnetic resonance imaging study; *n* = 24, one-sample *t*-test).

**Figure 2:**
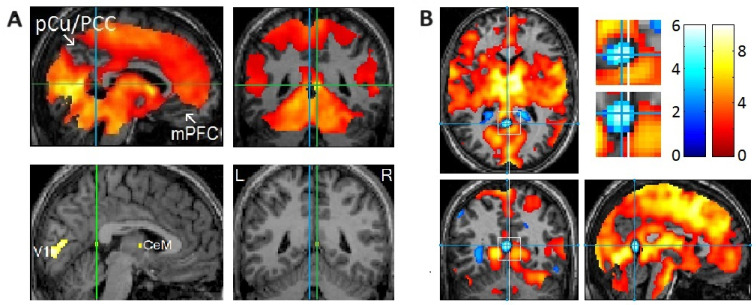
Rapid eye movement (REM)-locked deactivation in the retrosplenial cortex in the left hemisphere (RSC-Lt) and peak activation in the adjacent RSC in the right hemisphere (RSC-Rt). (**A**) Group analysis (*n* = 24). In the upper row, an uncorrected *p* < 0.05 was used to show areas of undetectable or attenuated activation. In the bottom row, the threshold was raised to a very high level of corrected *p* < 0.00005 (T = 10.1) to show the REM-locked peak activation. The green lines pass the RSC-Rt peak activity point (Talairach coordinates: 4, –46, 12, *t* = 10.5) and show the location of the sagittal view. The blue line passes the corresponding location in the contralateral hemisphere and shows the location of the sagittal view. PCu/PCC, precuneus/posterior cingulate cortex; mPFC, medial prefrontal cortex; CeM, central medial thalamic nucleus. (**B**) Individual study (one of six that showed an REM-locked functional magnetic resonance imaging (fMRI) signal decrease in the RSC-Lt). Cluster size = 86 voxels (voxel size = 2 mm × 2 mm × 2 mm). Blue lines pass the maximum fMRI blood oxygenation level-dependent signal decrease voxel. The white line indicates the mid-sagittal plane. Uncorrected *p* < 0.05. REM-locked deactivation in RSC-Lt was replicated in 6 of 24 independent individual studies. Adapted with permission from Hong et al. [20,25].

In addition to the clear localization of REM-locked peak activation in V1 (Figure 1A), peak activations were clearly localized in thin structures like the TRN and claustrum (Figure 1B), which are involved in multisensory–motor integration [51,52,53,54]. Notably, REM-locked activation also occurred in non-visual primary sensory cortices [20]. REM-locked peak activation occurred in the superior temporal gyrus (STG) in the left hemisphere (Figure 1B), which plays a central role in audiovisual integration [55,56]. Another surprising finding was the presence of REM-locked peak activation in the RSC-Rt and contrasting deactivation in the adjacent RSC in the left hemisphere (RSC-Lt) (Figure 2). This finding is remarkable because the RSC is relatively small, and the RSC-Rt and RSC-Lt border each other. REM-locked RSC findings are expounded on below. REM-locked peak activation was localized bilaterally in small areas overlapping the cholinergic basal nucleus, the major source of cholinergic input to the cortex, including the visual cortex [57]. Interestingly, cholinergic basal forebrain neurons in freely moving cats exhibited dramatically increased discharge rates (to the maximum rates) during movements in active waking and during active (phasic) REM sleep with bursts of REMs [57]. REM-locked peak activation in both the RSC-Rt and basal forebrain suggests REM-locked recruitment of the navigation system [58]. REM-locked activation in the cholinergic basal nucleus, which innervates the sensory cortex, may play a role in encoding, modulating, and gating predictive error signals [25,34]. Additionally, REM-locked peak activation was clearly localized in the BNST (Figure 1B).

To highlight peak REM-locked activations, the threshold was raised to the very high level of *p* < 0.00005, corrected for multiple comparisons (T = 10.1; A–C). (Figure 1A,B) Peaks were localized to the striate cortex, i.e., the primary visual cortex (V1). The boundary of V1 is denoted by yellow lines in the sagittal and coronal views in the lower images in (A); white arrows in (B) indicate V1. V2, extrastriate cortex; Cls, claustrum (the posterior–ventral zone, i.e., the putative visual zone); STG, superior temporal gyrus; BST, bed nucleus of stria terminalis; CeM, central medial thalamic nucleus. The thalamic reticular nucleus is indicated by green arrows. The green lines in (A) correspond to the locations of the other views. The green lines in (B) (coronal view inside the box) correspond to the locations of the lower four axial views. The number in the box denotes the distance (in mm) from the anterior commissure (A,C). The numbers in the axial views denote the distances (in mm) from the AC-posterior commissure plane. (Figure 1C,D) Areas of activation within the orthogonally oriented “glass brain”. (D) REM-locked activation is characteristically widespread (corrected *p* < 0.05). Adapted with permission from Hong et al. [20].

### 2.2. REM-Locked Peak Activation in the BNST

The REM-locked peak activity in the BNST has been puzzling. While preparing this review, I found a paper that provided me with new and coherent insights into REM-locked BNST activation. Lebow and Chen [59] posited that the BNST, sometimes referred to as the extended amygdala, performs valence surveillance of visual percepts, i.e., “the surveillance and assignment of valence to the information collected” regarding changes in the environment and social settings, including in food, mates, and infants, as well as threats. As an example of threat monitoring, an fMRI study noted activation of the BNST as participants viewed a video clip of a tarantula moving toward their feet [60]. From my new perspective, REM-locked “valence surveillance” in the BNST follows REM-locked processing of visual information and the generation of models of the bodily self and the world. Valence monitoring is essential for the interaction of the self with the world, including other people. Thus, an REM-locked peak in the BNST is relevant to studying problems with social communication and interaction in autism. Event-related fMRI can capture consecutive snapshots of fundamental mind/brain events, i.e., hierarchical generation of visual percepts in the dreaming brain [20], starting in V1; then, multisensory integration is accomplished in the TRN [51], claustrum [52,53,54], and STG [55,61], followed by “valence surveillance” of the generated visual percepts in the BNST [59].

### 2.3. The Role of the RSC in Spatial Processing

Crucially, we proposed that the RSC plays a role in generating the *spatial* bodily self-model (and placing it at the center of the world model) during dreaming and wakefulness [25]. The out-of-body illusion is experimentally induced by manipulating multisensory integration using head-mounted displays and synchronous visuotactile stimulation [43]. An fMRI study performed during an experimentally induced out-of-body experience suggested that the posterior cingulate cortex (PCC) and RSC play a key role in computing the location of the bodily self and head direction in spatial reference frames [62]. This study indicates that the RSC plays a key role in placing the bodily self-model at the center of the world model, and this centering mechanism can be experimentally manipulated or may go awry.

The RSC is “a hub between visual processing streams and the medial temporal lobe regions” that is essential for episodic memory, and the RSC is “at the top of a cortical visual perception hierarchy” [63]. This extensive synthetic review of the RSC assessed its roles in multiple sensorimotor and cognitive processes and grouped them into two categories: (1) perspective shifting across egocentric, allocentric, and route-centric spatial reference frames (“translating between perspectives”), and (2) serving as a critical node in the “predictive coding hierarchy” for the generation of internal representations of the environment (comparing perceptual inputs with memory).

The RSC’s role in the spatial navigation of the bodily self through the environment is well established [63]. The RSC also contributes to autobiographical, episodic memory, which is seemingly distinct from spatial navigation [64,65,66]. Scene construction underpins both autobiographical memory and spatial navigation [66,67,68]. Given that the brain constructs scenes when dreaming, the REM-locked activation of the RSC-Rt makes sense. Indeed, a quantitative meta-analysis of fMRI and PET studies provided evidence that the RSC subserves both autobiographical memory and navigation (and that the RSC-Lt constitutes the DMN) [69]. In line with this, spatial disorientation and/or episodic memory impairment are early signs of Alzheimer’s disease [70,71,72]. Additionally, RSC hypometabolism [73,74] and atrophy [75] have been found in the earliest stages of Alzheimer’s disease. Similarly, deficits in spatial navigation [76,77], episodic memory [76], and scene construction [78] were reported in autism (reviewed by [67]). It is assumed that REM-locked RSC activation/deactivation is different in autism.

### 2.4. Striking Contrast Between the RSC-Rt and the RSC-Lt

The compelling evidence for the lateralization of RSC function [20,21], along with other robust evidence for lateralization [64,79], requires explanation to incorporate lateralization into our concept thereof. Topographical disorientation and problems with spatial navigation follow RSC-Rt lesions in most case reports [64]. Episodic memory deficits were more apparent following lesions of the RSC-Lt [64].

Darby et al. (2017) studied 17 cases of delusional misidentification (“delusion of doubles” of others, places, pets, and inanimate objects familiar to the self) and related delusions about the bodily self (Cotard delusion). All cases had focal brain lesions. The authors mapped the lesion locations to a common brain atlas, and they showed that the RSC-Lt was the only site in the brain functionally connected to all 17 lesion locations; interestingly, the correlations were all negative. In other words, delusional misidentifications and Cotard delusion occurred when the areas inhibited by the RSC-Lt were damaged. Note that the RSC-Lt itself was not damaged in those 17 cases. The authors also showed (in a separate meta-analysis) that the RSC-Lt plays a key role in familiarity perception; the RSC-Lt was the region most likely to be activated by personally familiar (vs. unfamiliar) sensory (visual and auditory) stimuli (persons, places, and objects) [79]. It was suggested three decades earlier that delusion of doubles of familiar persons (the Capgras delusion, which is often accompanied by delusion of doubles of self), and of familiar inanimate objects, results from brain lesions disrupting affective familiarity with visual percepts [80]. The RSC-Lt appears to play a key role in attributing familiarity to visual percepts of the bodily self as well as objects in the world [79]. The brain must generate self-intimacy for the survival of the organism [40]. We take self-intimacy for granted, but the Cotard’s patient cannot consciously experience familiarity with himself [40]; persons with prosopagnosia cannot recognize familiar faces [80] or, in some cases, their own face reflected in a mirror [40]. In summary, evidence suggests that RSC-Lt controls―via tonic inhibition and permissive disinhibition―the process of generating and situating models of persons and objects familiar to the self in corresponding locations in spatial reference frames (with the bodily self model in the center, imbuing it with self-intimacy). Apparently, deactivation occurs only on one side (in the RSC-Lt) because the spatial reference frames should be kept active (by the RSC-Rt) during this spatial processing.

### 2.5. REM-Locked Activation Was Characteristically Widespread

REM-locked activation was characteristically widespread in our study (Figure 1D). Widespread REM-locked activation appears to be essential for generating dream consciousness [25], just as global brain activation is essential for generating waking consciousness. The global availability of incoming sensory information to multiple brain systems may be essential for conscious experience [40,81]. Active inference underlies conscious perception and requires a global neural workspace [82]; as REMs exemplify active inference, the REM-locked widespread activation that we established [20] makes sense. The generation of dreaming consciousness is an energy-intensive process that involves the whole brain. Brain-wide hyperemia during phasic REM sleep in rats [83,84] may also be time-locked to REMs, as expanded on below.

### 2.6. Relatively Small Islands of Attenuated REM-Locked Cortical Activation Were Restricted to the DMN

**Figure 3:**
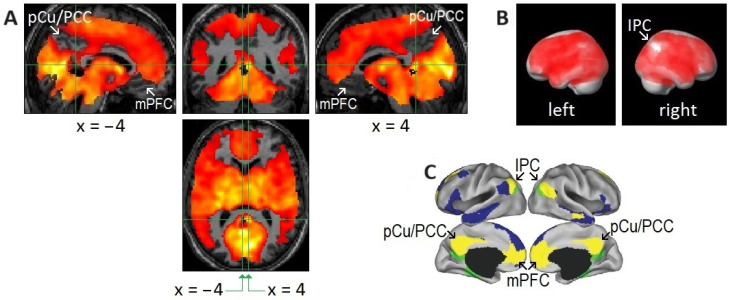
Areas of undetectable or attenuated rapid eye movement (REM)-locked activation. (**A**,**B**) Areas of undetectable or attenuated REM-locked activation correspond closely to the core of the default mode network (DMN) identified by a large-scale meta-analysis. An uncorrected *p*-value of <0.05 was used to identify areas of undetectable or attenuated activation in group-level random effects analysis (*n* = 24; one-sample t-test). The top and bottom parts of the brain are out of the field of view and thus truncated. (**A**) The precuneus/posterior cingulate cortex (PCu/PCC) and medical prefrontal cortex (mPFC) in the left and right hemispheres. Crosshair at the retrosplenial cortex in the right hemisphere (RSC-Rt) (Talairach coordinates: 4, −46, 12) and at the corresponding location in the contralateral hemisphere (Talairach coordinates: −4, −46, 12). The pocket of no activation in the retrosplenial cortex in the left hemisphere (RSC-Lt) contrasts dramatically with the adjacent robust activation in the RSC-Rt (*t* = 10.5). (**B**) Effects of REM-locked activation projected onto a surface rendering of a template brain. Note the absence of activation in the inferior parietal cortex (IPC) in the right hemisphere: there was attenuated activation of the IPC in the left hemisphere and bilateral deactivation in the inferior lateral temporal cortex and inferior frontal gyrus. (**A**,**B**) Adapted with permission from Hong et al. [21]. (**C**) Adapted with permission from the large-scale meta-analyses of Andrews-Hanna et al. [85]. DMN core (yellow), dorsal medial subsystem (blue), and medial temporal subsystem (green).

The relatively small cortical areas exempt from widespread REM-locked brain activation were restricted to the DMN in our study [21] (Figure 3). The DMN is a highly correlated network of brain regions comprising the medial prefrontal cortex, PCC, precuneus, RSC, posterior inferior parietal cortex, hippocampus, and parahippocampus [85,86,87]. This intrinsic brain network is activated by default during rest and when the brain is not attending to external sensory input [88]. It has already been observed that the DMN is deactivated when the brain is attending to exogenous sensory inputs. Deactivation of the DMN time-locked to REMs in sleep suggests that the DMN is also deactivated when the brain is attending to *endogenous* inputs as well [21]. The DMN at the top of a cortical hierarchy is distant from, and less tethered by, the sensorimotor system [89,90,91,92]. The intrinsic organization and reciprocal fluctuations in the “task-positive network” and DMN seen during wakefulness [93] persist in REM sleep [21,94].

Does the DMN simply rest, disengaged, during task performance? Evidence suggests otherwise, i.e., the DMN is involved in tasks [90,91,95,96,97] and provides top-down predictions to lower levels in the hierarchy [90,97,98,99]. We expanded this proposal by suggesting that the DMN at the top of a hierarchy may contextualize the generation of visual percepts by permissive disinhibition [21]. Permissive release of tonic inhibition underpins the control of saccadic eye movements: oculomotor neurons are tonically inhibited by omnipause neurons, which are themselves tonically inhibited by the superior colliculus (in turn tonically inhibited by the substantia nigra) [27]. Permissive disinhibition may be a general neuronal mechanism of oculomotor control. At the least, REM-probe fMRI studies can capture REM-locked brain responses from the bottom (V1) to the top (DMN) of the hierarchy.

## 3. REM-Locked Peak Activation Sites and Neural Processing Overlap with Atypicality in Autism

### 3.1. REMs in Sleep Are Saccades

It is relevant to autism research that REMs in sleep are saccades. Studies of saccadic eye movements in children with ASD have revealed that these children show differences in measurements of certain types of saccades compared with typically developing children [100,101,102]. This oculomotor dysfunction in ASD may serve as an early marker for ASD [100,103,104] and is innately related to social communication and social interaction [105,106]. As well as oculomotor dysfunction, superior visual search performance in infant siblings of children with ASD was associated with a subsequent ASD diagnosis and social interaction and communication [107,108,109].

Regarding visuospatial attention, which is coupled with oculomotor control, a meta-analysis of 38 eye-tracking studies showed that autistic individuals spend less time looking at social stimuli [110]. A recent large-scale eye-tracking study showed that toddlers (aged 12–48 months) with ASD direct more visual attention toward dynamic geometric images relative to dynamic social images, and this atypicality in ASD may be genetically driven [111]. Attention to the eyes and mouth is strongly influenced by genetic factors and is atypical during infancy and toddlerhood in autism [112]. Attention to eyes is present until it declines from 2 to 6 months of age in infants later diagnosed with autism, suggesting that there may be a window of opportunity for early intervention [113]. Accumulating evidence suggests the aberrant development of the visuospatial attentional network in ASD. The oculomotor system controls visuospatial attention, which is coupled with saccadic eye movements. REMs in sleep, being saccades, could aid in the study of the oculomotor system/visuospatial attentional network, including its atypical development in autism.

### 3.2. REMs Index Multisensory–Motor Integration

Meta-analyses indicated that autistic individuals showed impaired audiovisual integration, characterized by reduced susceptibility to the McGurk effect [114,115]. The McGurk effect is “seen as a proxy measure for multisensory integration” [19]. A majority of adults perceived [da] sounds when [ba] sounds were paired with video presentations of lip movements for [ga]; this illusion (the McGurk effect) is a classic demonstration of the audiovisual integration that occurs during speech perception [116].

Temporary disruption of the superior temporal sulcus with transcranial magnetic stimulation reduced the likelihood of observing the McGurk illusion [61]. The superior temporal sulcus was found to be both structurally and functionally atypical in ASD [117,118]. REM-locked peak activity occurred in the STG; interestingly, this was seen in the left hemisphere, i.e., the dominant hemisphere in speech in most people [20]. Notably, robust REM-locked activation occurred in Broca’s and Wernicke’s language areas [20]. Longitudinal REM-probe studies of infant siblings of children with ASD may shed light on the typical and atypical development of multisensory integration, particularly audiovisual integration and speech perception.

### 3.3. REM-Locked Peak Activation in the Cholinergic Basal Nuclei

The cholinergic system is one of the neurotransmitter systems implicated in autism. Nicotinic receptors in the cerebral cortex and cerebellum are extensively reduced (by 60–75%) in autism [119]. Voxel-based morphometry revealed regional gray matter reduction in the basal forebrain, cerebellar hemispheres, precuneus, and various other brain structures [120]. REM-probe studies may enable indirect investigation of neuronal firing of the cholinergic basal nucleus in autism.

### 3.4. REM-Locked Peak Activation in the BNST

The BNST, along with oxytocin, is involved in social vigilance and interactions [121,122]. The BNST expresses high levels of oxytocin receptors [123], and oxytocin and autism were part of a Bayesian predictive coding framework [124]. Indeed, the posterior BNST was implicated in social signaling deficits in male BTBR mice in an animal model of autism [125]. It is expected that REM-locked activation in the BNST would be reduced in an animal model of autism and in people with autism.

### 3.5. REM-Locked RSC-Rt Peak | RSC-Lt Deactivation

Among the DMN structures, an animal study highlighted the involvement of the RSC in particular in the development of ASD [126]; the authors identified a de novo mutation in the sentrin-specific peptidase1 gene in an ASD proband and demonstrated that haploinsufficient mice exhibit core autistic-like features, such as social deficits and repetitive behaviors, and that the agranular RSC is responsible for the core features of ASD in mice. Another recent animal model of ASD also highlighted the RSC. Shank2/3 double knockout within the RSC severely impaired social memory, which was rescued by designer receptors exclusively activated by designer drugs (DREADD)-mediated neuronal activation [127].

### 3.6. REM-Associated DMN Deactivation

Many studies found extensive overlap between the DMN and the “social brain”, i.e., the brain regions involved in social cognition (reviewed in [97]). The DMN is involved in the interaction between the self and the shared social world, that is, social cognition, social communication, and social interaction [97], deficits in which are core features of autism; this suggests a critical role of DMN in the development of autism.

Numerous studies have proposed a critical role of the DMN in connectivity atypicality in autism. Particularly relevant to the proposed REM-associated permissive deactivation by the DMN [21] are studies that have shown failure to deactivate the DMN during visual tasks in autism. An fMRI study showed a lack of DMN deactivation while a Stroop task was being performed by adults with autism; furthermore, the less deactivation of the medial prefrontal cortex, a DMN node, the higher the social impairment scores [128]. Adults with autism showed less deactivation of the DMN nodes while watching video clips in a theory of mind task [129]. Failure to deactivate the DMN during a visual search task was observed not only in adolescents with autism but also in their unaffected siblings, suggesting that failure to deactivate the DMN is a possible endophenotype of autism [130]. Reduced GABAergic inhibition in the early visual cortex may underpin the failure to deactivate the DMN during visual tasks in autism [8,131,132].

Given the DMN deactivation accompanying REM-locked visual network activation [21], studies of DMN–visual network connectivity in autism are relevant to REM-probe studies. An fMRI connectivity study of toddlers showed hypoconnectivity between the DMN and visual network during natural sleep in an autism subtype; approximately 20% of the toddlers with ASD spent most of their time looking at colorful geometric shapes (vs. adjacent videos of dancing children), and in those toddlers, DMN–visual network connectivity was inversely correlated with the Autism Diagnostic Observation Schedule social affect score [133]. In line with this finding, an IBIS study of infant siblings at high and low familial risk for ASD showed that the DMN–visual network connectivity at 12 months (during natural sleep) was inversely correlated with ritualistic/sameness and stereotyped behaviors [134]. An advanced connectome analysis of resting-state fMRI (rs-fMRI) data revealed significant reductions of connectome gradient scores in autism, primarily in the DMN, that is, overarching imbalances in network hierarchy between low-level sensory areas and the DMN at the top of the hierarchy [135].

Regarding the onset of the atypical connectivity seen in autism, a data-driven analysis of rs-fMRI connectivity in infant siblings of ASD probands showed that atypical hemispheric connectivity of the PCC (a hub of the DMN) and the extrastriate cortex emerges within the first year of life [136].

It is expected that REM-locked DMN deactivation would not be observed in children with autism or their unaffected siblings. This atypical connectivity may be observed within the first year of life. As much of the external sensory input to the brain is blocked in REM sleep [137], REM sleep may be an ideal state to study the intrinsic organization of large-scale brain networks. REM-probe studies in infant siblings may advance knowledge of typical and atypical developmental trajectories of the intrinsic organization of large-scale brain networks (particularly the anticorrelation between the DMN and visual processing network).

## 4. Strengths of REM-Probe Studies

### 4.1. REMs Are a Task-Free Probe

The strengths of REMs as a probe in functional brain imaging studies are manifold, particularly during infancy. REMs serve as *a task-free probe* useful for studying both infants and animals, who cannot comply with conventional visual activation tasks or report conscious experience. As declared in a recent publication, “Infants and animals are … unable to produce introspective reports [of conscious experience]” and “there is a pressing need to identify ‘markers’ of [their] consciousness” [138]. REMs in sleep occur in mammals and birds [23], and even in Australian dragons [139]. REM-locked neural changes can be studied in species other than humans, such as Australian dragons [139]. Employing REMs in sleep as a natural probe aids in the design and establishment of a standardized set of sensory paradigms suitable for cross-species comparison [8]. REMs in sleep may allow straightforward comparison of, and translation between, animal and human data. Thus, REM-probe studies of animal models of autism have great potential.

### 4.2. The Level of Attention Is Controlled

Additionally, differences in the level of attention confound comparisons across the lifespan and between individuals with and without autism. In an REM-probe study, the level of attention across the lifespan, and across typical and atypical development, is controlled for. Thus, REMs in sleep are a useful probe for studying lifelong neurodevelopmental disorders, like autism, across the lifespan.

### 4.3. The Brain Is Relatively Isolated from the Environment

REM sleep is an ideal state to study the intrinsic organization of large-scale brain networks (including the DMN) because the brain is relatively isolated from the environment [137,140,141,142]. In studies of visual perception in wakefulness, perturbation by irrelevant non-visual sensory signals arising from the environment may confound the interpretation of empirical findings [143]. The brain inhibits not only sensory input but also motor output during REM sleep. Muscle atonia occurs during REM sleep, limiting head movements, which can create data artifacts in MRI studies.

### 4.4. Sufficient Statistical Power for Longitudinal, Within-Individual Analysis

In an individual-level analysis, all 24 studies, including a short (6 min) study with 43 REMs, showed similar regional patterns of activation and deactivation, including robust REM-locked activation in the V1 and REM-locked periventricular signal decrease (PVSD) [20]. To achieve the aim of early detection, prevention, and improvement of long-term outcomes [4,5], longitudinal studies using a method with sufficient statistical power for within-individual analysis are essential. The statistical efficiency of REM-probe studies may facilitate the aims of IBIS “to predict *on an individual basis* an ASD diagnosis” and “[move] beyond group-level differences toward individual-level prediction” [4].

### 4.5. The Preponderance of REM Sleep in Late Pregnancy and in Infancy

The preponderance of REM sleep in the last trimester of pregnancy, and after birth [22,23], makes infants ideal participants for REM-probe studies. REM-probe studies will be a great advantage for research on the infant siblings of children with autism. Furthermore, fMRI and other brain imaging modalities have been used to study the development of large-scale brain networks in the fetus [144]. REM-probe fMRI studies are now feasible, even in the fetus. REMs were timed by identifying the center of the lens and eye on serial fMRI images, and an fMRI study of the neural correlates of REMs was performed in the human fetus [145]. In principle, longitudinal REM-probe studies are now possible from before birth.

### 4.6. Advantages over Traditional rs-fMRI Studies

rs-fMRI studies can also be performed during sleep. Do REM-probe studies have any advantages over traditional rs-fMRI studies? One potential advantage is that REM-probe studies enable the study of precise temporal changes time-locked to REMs, whereas rs-fMRI studies analyze data in a block of time (minutes). Time-series analysis of REM-locked hierarchical processing over time may be more suitable for investigating perception, which is a temporal process [108]. This is clearly illustrated by our event-related fMRI study of visual processing time-locked to REMs [20]. Additionally, rs-fMRI connectivity studies across species, described as “emerging approaches” of “critical importance” [146], may be complemented by REM-probe studies across species, considering that REM-probe studies capture serial snapshots of large-scale brain networks in an ideal, isolated brain state and that REMs allow straightforward comparison across species.

## 5. Functional Ultrasound (fUS) Study of Animals and Human Infants

A group used ultrasound technology to develop fUS [147,148], a new, fully fledged brain imaging technique with far superior spatiotemporal resolution to fMRI. They subsequently used fUS to study the time course of cerebral blood volume changes during phasic REM sleep in rats [83,84]. fUS studies of REM sleep in rats revealed interesting similarities with REM-locked findings in humans. They found massive brain-wide cerebral blood volume spikes lasting 5–30 s during phasic REM sleep and called them “vascular surges” [84]. Notably, the time course of the widespread cortical “vascular surges” during phasic REM sleep in rats [83,84] is similar to that of the widespread brain activation time-locked to REMs occurring during phasic REM sleep in humans [20]. Thus, the vascular surges seen in rats during phasic REM sleep may also be time-locked to REMs. The use of a close-up camera to time REMs during fUS was then proposed [83], as I also suggested. As an additional similarity, sensory processing was coupled with deactivation of the RSC both in humans (only in the RSC-Lt) [20] and in mice (in the RSC-Lt, more so than in the RSC-Rt) [149].

The most consistent and robust REM-locked blood oxygenation level-dependent (BOLD) signal decrease was found in the periventricular areas [20]. PVSD occurred in all of our 24 individual studies. Interestingly, BOLD signal decreases around large cerebral veins in periventricular areas, anticorrelating with visual cortex activation, were found in the waking resting state using a 7-T scanner [150]. This study suggests that REM-locked PVSD reflects vasodilation rather than neuronal deactivation [21,150]. Notably, fUS studies of REM sleep in rats [83,84] observed vasodilation in the whole brain. It is unlikely that REM-locked vasodilation occurs only in the periventricular areas in humans. Rather, it is likely that fMRI can detect vasodilation only at the water–brain tissue border (because of the partial volume effect in fMRI), even though vasodilation occurs brain-wide in humans as well as rats. Notably, REM-locked PVSD, found in all 24 adults, was not found in the neonate who participated in our feasibility study.

fUS has high sensitivity and a temporal resolution of 10–100 ms [147]. fUS enabled the assessment of the top-down propagation of signals in hierarchical processing after only a single trial of visual tasks [151]. Studying hierarchical REM-locked processing of endogenous visual signals [20] using fUS will expand our knowledge regarding the generation of dreaming and waking consciousness, given the high resolution. Crucially, fUS REM-probe studies of animal models of autism have great potential in autism research. Notably, this group performed trans-fontanel (where the skull bone that impedes fUS is absent) fUS imaging in human neonates during sleep [152,153]. As the portability of fUS enables bedside monitoring of neonates, it has great potential in ASD infant sibling studies.

## 6. Practical Considerations in Conducting an REM-Probe Study

In fMRI studies of the neural correlates of REMs, the timing of REMs is as crucial as the fMRI measurements themselves. REMs should be timed from video recordings instead of electrooculograms (EOGs), the conventional method for monitoring eye movements. Rapidly changing magnetic fields during MRI confound the EOG signal. Thus, MRI scanner artifacts need to be removed by a filter [154,155]. Filtering reduces the number of eye movements detected, especially small-amplitude eye movements. Note that small and large eye movements may have almost the same effect on the fMRI signal [156]. Video monitoring detected approximately four times as many REMs as EOG in fMRI studies [20]. The video timing of REMs revealed a series of surprising findings that were not seen with EOG timing of REMs. Note that eye movements detected by video monitoring and EOG are fundamentally the same. EOG measures electric currents induced by the movements of the electrically charged eyeball, whereas video monitoring directly measures eye movements. Indeed, we showed that the REM timings derived from EOG and video recordings were in excellent agreement [20]. For a study of the neural substrates of REMs that does not aim to compare REM sleep with non-REM sleep, sleep staging on the basis of EOGs and electroencephalograms is not necessary.

How does REM sleep latency change across the lifespan? This question will arise when designing an REM-probe study across the lifespan. REM sleep latency increases linearly as newborns age, reaches its maximum (approximately 200 min) at ages 6–7 years [157,158], and then gradually decreases to approximately 100 min by age 18 years [158,159] and 60 min by age 80 years [159]. The long REM sleep latency of children will probably require two consecutive overnight assessments for REM-probe studies, as in our REM-probe studies in adults [20].

We showed the feasibility of REM-probe fMRI studies in neonates. We studied two neonates in 2003 to check the feasibility of an REM-probe study of neonates. The study was approved by the Institutional Review Board of Johns Hopkins Medicine. Informed consent was obtained from the parents of the infants. Eye movements were monitored and recorded using a video camera (video recordings of REMs of a neonate can be found in Supplementary Videos). fMRI data were obtained every 2 s using a Philips Gyroscan 1.5-T scanner (Phillips, Amsterdam, The Netherland) (20 slices; slice thickness = 4 mm) and a blipped echo-planar imaging-based gradient-refocused echo sequence. We obtained fMRI data as soon as REMs were recognized by video monitoring and continued recording for as long as they persisted. MRI scanners create a loud noise. Therefore, we implemented three noise-protection measures: ear plugs, MiniMuffs Noise Attenuators (Natus, Middleton, WI, USA), and headphones. The first neonate participant did not have enough REM sleep during the 2 h scan for analysis. The second participant had two REM sleep episodes, which were combined for the analysis. The total duration of the two REM sleep episodes combined was approximately 20 min. Sections with head movements were not included in the analysis. The eye movements were timed from the video recordings in exactly the same way as the REMs of adults. Statistical parametric mapping event-related analysis was performed to assess regional patterns of activation and deactivation in relation to the occurrence of REMs. The analysis revealed activation in the thalamus, and deactivation in the occipital cortex, time-locked to REMs. The deactivation in the occipital cortex was consistent with the signal decrease seen therein during visual stimulation in sleeping infants [160]. REM-locked PVSD was not found in the neonates that we studied. Note that REM-locked PVSD was observed in all 24 within-individual studies in adults.

Neonates are ideal participants for REM-probe studies because they sleep for 16–18 h per day, REM sleep is typically the initial sleep state, and more than half of the sleep time is spent in REM sleep until 2–3 months of age [161]. Whereas the MRI scanner had to be reserved for 2 consecutive nights for REM-probe studies in adults, it was reserved for only 2 h during the daytime for REM-probe studies in neonates. Optimizing data collection and analysis may further reduce scan duration in neonate studies, given the statistical efficiency of fMRI REM-probe studies in adults.

## 7. Limitations of REM Probe Approach

- In 6 of 11 adult participants in our study, a sufficient number of REMs occurred only on the second night. Presumably, head restraint, which is required for MRI studies, suppresses REM sleep, and REM sleep deprivation during the first night likely builds “REM pressure” for the second night [20]. This is a limitation of the REM-probe approach; two consecutive overnight studies are labor intensive, and it is costly to use an MRI scanner for two nights.
- Timing REMs by visual inspection is also labor-intensive. However, video recordings of closed eyes can be coupled with computerized analysis to time and quantify REMs automatically, as was done with REMs of Australian dragons [139].
- Timing REMs by visual inspection may be difficult for some people. We had difficulty timing REMs using video recording in 1 of the 14 participants [20].
- Out of the fourteen adult participants, one could not fall asleep and withdrew. Another participant had unusually large and frequent, jerky head movements and was not included in the analysis [20].
- We have limited experience in timing REMs in infants and no experience in timing REMs in animals. As the REM probe approach will lead researchers into uncharted territory, and alongside many surprising findings, we expect some challenges.

## 8. Conclusions

Timing REMs in sleep can benefit studies of infant siblings of children with ASD, which are usually performed during natural sleep. REMs in sleep are saccades that index the processing of endogenous visual signals and hierarchical multisensory processing. REMs are a prime example of active inference. Employing REMs as a task-free probe of infant visual brain development has far-reaching implications, and it may provide valuable data on the typical and atypical development of visual perception and multisensory–motor integration. It is expected that REM-locked activities may be atypical in autism in the oculomotor system, multisensory–motor integration, cholinergic basal nuclei, BNST, and RSC. Additionally, failure to deactivate the DMN in relation to REMs is expected in autism.

However, the main advantage of this paradigm for autism research lies in the exploration of uncharted territory, i.e., potentially capturing consecutive snapshots of large-scale brain networks at the moment of the generation of visual percepts in an ideal, isolated brain state. Our REM-probe PET study [26] led to the serendipitous finding that REMs in sleep may be saccades, and REM-probe fMRI studies in adults [20] yielded a series of surprising findings. Important insight regarding the implications of our fMRI findings were obtained approximately 5 years after the publication of the study, owing to the protoconsciousness theory of Allan Hobson and the predictive coding/active inference perspectives of Karl Friston; 14 years later, in the context of valence surveillance occurring in the BNST. Likewise, REM-probe fMRI or fUS studies comparing infants who develop autism with typically developing infants may lead to novel and enlightening findings regarding the typical and atypical ontogenetic development of visual perception and sensorimotor integration. fUS REM-probe studies of animals will shed light on the phylogenetic development of consciousness. In particular, fUS REM-probe studies of autism animal models may advance knowledge about autism. REMs in sleep are a uniquely promising marker of typical and atypical consciousness, particularly autism.

## Data Availability

The raw data supporting reported results will be made available by the corresponding author, without reservation.

## Supplementary Materials

The following supporting information can be downloaded at: https://www.mdpi.com/article/10.3390/brainsci15060574/s1, Video S1: Video recording of REMs of a neonate during our fMRI study.mp4. https://drive.google.com/file/d/0BzWLE2tIe0XhYXlBRnNRRHYtM2c/view?usp=sharing. From Hong et al. (2018) [25] with permission from Hong; Video S2: Video recording of REMs of an adult during our fMRI study.avi. https://drive.google.com/file/d/0B0R_1dukpEw7QXpObTNZLWp0M3M/view?usp=sharing. From Hong et al. (2018) [25] with permission from Hong.
